# LOX Expression and Functional Analysis in Astrocytomas and Impact of *IDH1* Mutation

**DOI:** 10.1371/journal.pone.0119781

**Published:** 2015-03-19

**Authors:** Roseli da Silva, Miyuki Uno, Suely K. Nagahashi Marie, Sueli M. Oba-Shinjo

**Affiliations:** 1 Laboratory of Molecular and Cellular Biology, Department of Neurology, Faculdade de Medicina da Universidade de São Paulo, São Paulo, 01246-903, Brazil; 2 Center of Translational Research in Oncology, Instituto do Câncer do Estado de São Paulo (ICESP), 01246-000, São Paulo, Brazil; 3 Center for Studies of Cellular and Molecular Therapy (NETCEM), University of São Paulo, São Paulo, Brazil; Sun Yat-sen University Medical School, CHINA

## Abstract

Lysyl oxidase (LOX) is involved in vital biological processes such as cell motility, cell signaling and gene regulation. Deregulation of this protein can contribute to tumor formation and progression. Although it is known that LOX is involved in invasion, proliferation and tumor migration in other types of tumors, studies of LOX in astrocytomas of different grades are scarce. The purpose of our study was to characterize *LOX*, *BMP1* and *HIF1A* expression by real-time PCR in astrocytomas with WHO grades I to IV compared to non-neoplastic brain tissue. *IDH1* mutational status was determined by PCR and sequencing. LOX protein expression was also analyzed by immunohistochemistry. LOX functional analyses were performed using siRNA knockdown and the specific inhibitor BAPN in two glioblastoma cell lines. The expression levels of *LOX*, *BMP1* and *HIF1A* were correlated and analyzed according to *IDH1* mutation status and to the clinical end-point of overall survival of glioblastoma patients. The results demonstrate that increased expression and activity of *LOX*, *BMP1* and *HIF1A* were positively correlated with the malignant grade of astrocytomas. LOX protein expression also increased according to the degree of malignancy, with localization in the cytoplasm and nucleus and staining observed in endothelial cells. Glioblastoma with a mutation in *IDH1* expressed lower levels of LOX in the nucleus, and *IDH1*-mutated cases showed lower *LOX* expression levels when compared to wild-type *IDH1* cases. *LOX* knockdown and inhibition by BAPN in U87MG and A172 cell lines affected migration, invasion and soft agar colony formation. Taken together, these results corroborate the role of LOX in the migration, invasion and angiogenesis of astrocytomas. Furthermore, *LOX* expression is influenced by *IDH1* mutational status. This work provides new insights for researchers aiming to design targeted therapies to control astrocytomas.

## Introduction

Astrocytomas are the most common primary brain tumors. The World Health Organization (WHO) classifies astrocytomas into four malignant grades: grade I, or pilocytic astrocytoma; grade II, or low-grade astrocytoma (AGII), grade III, or anaplastic astrocytoma (AGIII); and grade IV astrocytoma or glioblastoma (AGIV or GBM) [1]. Diffusely infiltrative astrocytomas (AGII-GBM) have the ability to invade the surrounding normal brain tissue, hampering tumor resection. GBM, the most malignant and frequent brain tumor in adults, can be divided into two subgroups: primary GBM, which arises *de novo*, and secondary GBM, which results from the progression of a lower grade astrocytoma [2,3]. Interestingly, mutations in the gene that encodes isocitrate dehydrogenase 1 (IDH1) have been reported in diffuse gliomas, including WHO grades II and III astroglial and oligodendroglial lineages [4–8]. *IDH1* mutations are strong predictors of a more favorable prognosis and serve as a highly selective molecular marker of secondary GBM that complements clinical criteria for distinguishing secondary GBM from primary GBM [9,10,11].

Lysyl oxidase (LOX), a copper-dependent amine oxidase, catalyzes the enzymatic stage of collagen and elastin cross-linking by oxidizing primary amines into reactive aldehydes. These reactions are essential for stabilization of collagen fibrils and for the integrity and elasticity of mature elastin to ensure normal functionality of connective tissue, embryonic development and adult tissue remodeling [12]. Importantly, biologically active compounds, hydrogen peroxide and ammonia are generated as by-products during these catalytic reactions. LOX also has intracellular functions and is involved in the regulation of cell differentiation, motility/migration and gene transcription. Aberrant expression of the *LOX* gene has been reported in multiple tumors [13].

LOX is synthesized by several cell types as a 48 kDa protein. After signal peptide cleavage and N-glycosylation, the resulting 50 kDa proenzyme is secreted and converted into a mature, active 30 kDa form as a result of proteolytic processing by procollagen C proteinase/bone morphogenic protein-1 (BMP1). The catalytic activity of *LOX* can be specifically and irreversibly inhibited by beta-aminopropionitrile (BAPN) [14]. LOX has been identified as an important regulator of the hypoxia-induced tumor progression pathway through a HIF-1α-dependent mechanism in numerous cancer types, such as breast, head and neck, prostate and renal cell carcinomas [15,16,17]. LOX is involved in the hypoxic upregulation of *HIF1A*, and LOX and HIF-1α potentiate each other to foster tumor progression in the colon through the PI3K-Akt signaling pathway [15,18]. Secreted LOX is responsible for the invasive properties of hypoxic cancer cells, including astrocytomas, through the activation of focal adhesion kinase (FAK)/paxillin [19]. LOX has been implicated in tumor angiogenesis *in vitro* and *in vivo* by increasing vascular endothelial growth factor (VEGF) expression and secretion as well as blood vessel formation [20].

Recently, it was demonstrated that HIF-1α-responsive genes essential for cell growth, including *LOX*, were underexpressed in gliomas with *IDH1* mutation [21]. Therefore, we aimed to investigate *LOX*, *BMP1* and *HIF1A* mRNA expression levels in a large series of astrocytomas of different malignant grades and compare these results between cases with wild type *IDH1* and cases with mutated *IDH1*. *LOX* knockdown by siRNA was performed for functional studies *in vitro*, and LOX protein was also analyzed in tumor samples. These data suggest that *LOX* expression increases according to malignancy grade in astrocytomas and represents a potential therapeutic target, especially for cases without *IDH1* mutation.

## Methods

### Tissue Samples

The samples used in this study consisted of 153 astrocytomas (grades I to IV). Tumors were graded according to the WHO classification into AGI (n = 23; mean age at diagnosis, 19.4±9.7 years; 14 males and 9 females), AGII (n = 26; mean age at diagnosis, 34.0±8.1 years; 15 males and 11 females), AGIII (n = 18; mean age at diagnosis, 35.0±12.3 years; 11 males and 7 females) and GBM (n = 86; mean age at diagnosis, 54.0±13.9 years; 58 males and 28 females). The non-neoplastic control group consisted of samples from individuals undergoing temporal lobe resection during epilepsy surgery (n = 22; mean age at diagnosis, 38.0±7.6 years; 10 males and 12 females). All samples were collected during surgical procedures by the Neurosurgery Group of the Department of Neurology at the Hospital das Clinicas of School of Medicine, University of Sao Paulo, Brazil. Fresh surgical samples were immediately snap-frozen in liquid nitrogen upon surgical removal. Before RNA extraction, a 4-μm-thick cryosection of each sample was stained with hematoxylin, and necrotic and non-neoplastic areas were removed from the frozen block of tumoral tissue by microdissection. Grey matter was avoided in the control non-neoplastic samples. Written informed consent was obtained from all patients according to the ethical guidelines approved by the Ethics Committee of the School of Medicine, University of São Paulo (0600/10). The Ethical Commission for Research Projects Analysis (CAPPesq) from the Clinical Board of Hospital das Clinicas and School of Medicine, University of São Paulo, in council session taken place at 2012, August 8th, approved the research protocol entitled: “Expression and role of lysyl oxidase family genes in astrocytomas”, presented by the Department of Neurology.

### Total RNA extraction and cDNA synthesis

Total RNA was extracted from frozen tissues (tumor and non-neoplastic) using an RNeasy Mini kit (Qiagen, Hilden, Germany). The RNA concentration and purity were evaluated by measuring the absorbance at 260 and 280 nm. A 260/280 ratio ranging from 1.8 to 2.0 was considered satisfactory purity. Denaturing agarose gel electrophoresis was used to assess the quality of the samples. A conventional reverse transcription reaction was performed to yield single-stranded cDNA. First-strand cDNA was synthesized from 1 μg of total RNA that was previously treated with 1 unit of DNase I (FPLC-pure, GE Healthcare, Uppsala, Sweden) using random and oligo(dT) primers, RNase inhibitor, and SuperScript III reverse transcriptase according to the manufacturer’s recommendations (Life Technologies, Carlsbad, CA). The resulting cDNA was subsequently treated with 1 unit of RNase H (GE Healthcare, Uppsala, Sweden), diluted with TE buffer, and stored at −20°C until later use.

### Quantitative real time PCR (RT-qPCR)

The relative expression levels of *LOX*, *HIF1A* and *BMP1* were analyzed by RT-qPCR using the SYBR Green approach. Quantitative data were normalized to the geometric mean of three reference genes suitable for the analysis: hypoxanthine phosphoribosyltransferase (*HPRT*), beta glucuronidase (*GUSB*) and TATA box binding protein (*TBP*). The primer sequences were as follows (from 5’ to 3’): LOX F: CCTACTACATCCAGGCGTCCA; LOX F R: CATAATCTCTGACATCTGCCCCTGT; HIF1A F: CATCCAAGAAGCCCTAACGTGT; HIF1A R: CATTTTTCGCTTTCTCTGAGCAT; BMP1 F: CTCGTAAGTCCTCCATCAAAGCT; BMP1 R: CTCTCCATCTCCCACAGGCTC; HPRT F: TGAGGATTTGGAAAGGGTGT; HPRT R: GAGCACACAGAGGGCTACA; GUSB F: GAAAATACGTGGTTGGAGAGCTCATT, GUSB R: CCGAGTGAAGATCCCCTTTTTA; TBP F: AGGATAAGAGAGCCACGAACCA, TBP R: CTTGCTGCCAGTCTGGACTGT. The primers were synthesized by IDT (Integrated DNA Technologies, Coralville, IA, USA). The minimum primer concentrations necessary were determined to give the lowest threshold cycle (Ct) and maximum amplification efficiency while minimizing non-specific amplification. The primer concentrations used were 200 nM for *LOX*, *HIF1A*, *BMP1*, *HPRT* and *TBP* and 400 nM for *GUSB*. Standard curves were established to ensure amplification efficiency, and an analysis of melting curves demonstrated a single peak for all PCR products. Additionally, agarose gel electrophoresis was employed to check the size of the PCR products amplified. SYBR Green I amplification mixtures (12 μl) contained 3 μl of cDNA, 6 μl of 2x Power SYBR Green I Master Mix (Life Technologies) and primers. The PCRs were run on an ABI Prism 7500 sequence detector (Life Technologies) as follows: 2 min at 50°C, 10 min of polymerase activation at 95°C, and 40 cycles of 15 s at 95°C and 1 min at 60°C. Quantitative data were normalized relative to the internal housekeeping control genes. The equation 2^−ΔCt^ was applied to calculate the expression of *LOX*, where ΔCt = Ct of the target gene—geometric mean of the Ct of the reference genes [22]. The RT-qPCR reactions were performed in duplicate for each sample and repeated when the Ct values were not similar. The results are presented on a log10 scale for better visualization. *LOX* expression was scored according to the median expression values of each astrocytoma grade. For statistical analysis, scores equal to or higher than the median values were defined as *LOX* overexpression. For functional analysis of *LOX* knockdown after siRNA transfection, the same procedures were followed, except that only *HPRT* was used as a reference gene. The expression values were calculated relative to the scrambled non-target control (NTC).

### DNA extraction and IDH1 mutational analyses

DNA was extracted from frozen tumor samples using a QiaAmp DNA Micro kit (Qiagen). Polymerase chain reaction (PCR) followed by DNA sequencing was applied to detect mutations in IDH1, as previously described [23]. The sequences of primers (5’-3’) synthesized by IDT for PCR amplification of exon 4 were as follows: CCATCACTGCAGTTGTAGGTT and CATACAAGTTGGAAATTTCTGG. PCR products were generated in a 25 μL reaction mixture including 100 ng of DNA, 50 mM KCl, 50 mM of each dNTP, 10 mM Tris-HCl (pH 9.0), 1.5 mM MgCl_2_, 10 pmol of each primer and 1 unit of Taq DNA polymerase (GE Healthcare). The PCR was performed with an initial denaturating step at 94˚C for 5 min, followed by 35 cycles consisting of 94˚C for 30 s, 54˚C for 30 s and at 72˚C for 30 s. After the final cycle, an extension period of 10 min at 72˚C was performed. The PCR products (436 bp) were purified with a GFX column (GE Healthcare) and sequenced on an ABI Prism 3130 DNA automated sequencer using the Big Dye Terminator Cycle Sequencing Ready Reaction Kit version 3.1 (Life Technologies). The primers used for sequencing were the same as those used for PCR.

### Cell culture conditions and transient transfection

The human malignant astrocytoma cell lines U87MG and A172 were routinely cultured in Dulbecco’s modified Eagle’s medium (DMEM) (Life Technologies) supplemented with 10% fetal bovine serum (FBS) (Life Technologies), 100 IU/ml penicillin and 100 μg/ml streptomycin in an atmosphere consisting of 5% CO_2_ in air at 37°C in a humidified incubator. Dicer substrate small interfering RNA (siRNA) duplexes for *LOX* knockdown (5′-GUAAUUACAGAAUUGAAACACUGUGUU-3′) were diluted in buffer according to the manufacturer’s recommendations (IDT). U87MG and A172 cells (1 × 10^5^ cells/well) were seeded in a six-well plate; after 24 h, they were transfected with Lipofectamine RNAiMax (Life Technologies). Control cells were transfected with a scrambled non-target control (NTC) siRNA from IDT. siRNAs for both LOX and NCT were used at a concentration of 1 nM, and *LOX* knockdown as well as the effect of LOX silencing were evaluated at 2, 4, and 7 days after transfection by RT-qPCR and Western blotting.

### Cell migration and invasion assay

The migration and invasion abilities of the cells were assessed by determining the ability of the cell lines to cross a membrane with a pore size of 8 m in non-coated (for migration) and matrigel-coated (for invasion) Transwells (BD Biosciences, Becton, Dickinson and Company, Franklin Lakes, NJ), according to the manufacturer's recommendations. After 48 hours of transfection, the cells were maintained in DMEM supplemented with 1% FBS for 2 hours. To determine the effect of LOX inhibitor treatment on migration, the cells were treated with 100 mM β-aminopropionitrile (BAPN Sigma-Aldrich, Saint Louis, MO) for 24 h prior to the migration assays and for an additional 4 h during the assay. This concentration of BAPN has no cytotoxic effects, as demonstrated previously [24]. After trypsinization, a total of 2.5 x 10^4^ cells were suspended in 0.5 ml DMEM with 1% FBS and added to the upper compartment of the Transwell. The bottom chamber contained 10% FBS, and the cells were incubated for 18 hours at 37°C in an atmosphere containing 5% CO_2_. Non-migrating and non-invading cells were wiped away from the upper surface of the membrane with a cotton swab. The cells were fixed with 4% paraformaldehyde, stained with 0.2% crystal violet (Sigma-Aldrich) in 20% methanol, and analyzed by inverted microscopy (40x magnification). The results of the migration and invasion assays were quantified by counting 18 random fields from each of the experimental inserts in duplicate. Data were generated from two independent experiments. Migration values were expressed as the average number of migrated cells per microscope field.

### Cell proliferation and anchorage-independent cell growth assay

Proliferation of U87MG and A172 cells was evaluated after 2, 4, and 7 days of siRNA *LOX* transfection in duplicate experiments. The cells were stained with Trypan blue and counted using an automatic counter (Countess, Life Technologies) for determination of live cells. Anchorage-independent cell growth was analyzed via a soft agar colony formation assay. Two days after transfection, 1 x 10^3^ cells were resuspended in 0.3% agar on a layer of 0.6% agar in DMEM (1x) in a six-well plate and incubated in a humidified atmosphere in the presence of 5% CO_2_ at 37°C. After 10 days, the colonies were fixed with formaldehyde and stained with 0.005% crystal violet in phosphate-buffered saline (PBS) for 1 h. The number of colonies was recorded for each well. Two independent experiments were performed in duplicate.

### Western blot analysis

After transfection with control siRNA (NTC) and siRNA specific for *LOX*, total protein lysates were prepared from cell cultures with RIPA lysis buffer and protease inhibitor cocktail (Sigma-Aldrich) on ice. The protein concentration was determined using the Bradford reagent (Bio-Rad Laboratories, Richmond, CA). The absorbance was measured at 595 nm using a microplate spectrophotometer (Multiskan Spectrum; Thermo Labsystems, Helsinki, Finland). All assays were conducted in duplicate, and calculations were carried out with a standard curve constructed using different concentrations of bovine serum albumin (2 mg/ml to 0.125 mg/ml). Total protein lysates (30 mg) were separated by 12% SDS polyacrylamide gel electrophoresis (TGX Mini Protean, Bio-Rad) with Tris-glycine running buffer. The proteins were transferred to a nitrocellulose membrane using the iBlot dry blotting system (Life Technologies). The membrane was blocked with 5% skim milk and incubated with rabbit polyclonal primary anti-LOX diluted 1:1,000 (Sigma-Aldrich). The membrane was also incubated with mouse monoclonal anti-β-actin (1:5,000, clone AC-74, Sigma-Aldrich) as a protein loading control. The secondary antibodies used were anti-rabbit (1:1,000) and anti-mouse IgG (1:5,000) conjugated to peroxidase (Sigma-Aldrich). The immune complexes were visualized using enhanced chemiluminescence reagent (Western Lightning Chemiluminescence Reagent Plus, Perkin Elmer, Waltham, MA) and detected with ImageQuant LAS4000 (GE Healthcare).

### Immunohistochemistry

For immunohistochemical detection, 4-μm formalin fixed paraffin embedded tissue sections were routinely processed and subjected to antigen retrieval. Briefly, slides were immersed in 10 mM citrate buffer (pH 6.0) and incubated at 122°C for 3 min using an electric pressure cooker (BioCare Medical, Walnut Creek, CA). Specimens were then incubated for endogenous peroxidase blocking (Novolink system, Novocastra, Newcastle-upon-Tyne, UK) and further incubated with a polyclonal antibody raised in rabbits against human LOX (ab31238, 1:100 dilution; Abcam, Cambridge, UK) at 16–20°C for 16 hours. The reaction was developed with a commercial kit (Novolink; Novocastra, Newcastle-upon-Tyne, UK) at room temperature using diaminobenzidine and Harris hematoxylin for nuclear staining. Optimization using a positive control suggested by the manufacturer (breast carcinoma) was performed to obtain the optimal dilution. The staining intensity of tissue sections was evaluated independently by two observers (SKNM and RS). A semi-quantitative scoring system considering both the intensity of staining and percentage of cells was applied as follows: for intensity of staining, 0 = negative, 1 = weak, 2 = moderate and 3 = strong; for cell percentage, 0 = no cells stained, 1 = 10–25%, 2 = 26–50%, 3 = 51–75% and 4 = 76–100%. The LOX immunohistochemistry labeling score (ILS) was obtained by determining the product of the staining intensity and the percentage of cells stained. Digital photomicrographs of representative fields were captured and processed using PICASA 3 (Google, USA).

### Statistical analysis

Kolmogorov-Smirnov normality test was used to analyze the distribution of gene expression data and also the effect of *IDH1* mutations on gene expression. The Kruskal- Wallis test was used for analysis of the differences in gene expression between non-neoplastic tissues and astrocytomas of different grades of malignancy. Coexpression of genes was analyzed using the Spearman-rho test. A correlation coefficient (r) of ≥0.7 was interpreted as a strong correlation, 0.3≤r<0.7 was interpreted as a moderate correlation, and r <0.3 was interpreted as a slight correlation. The Mann-Whitney test and *t* test were used to compare *IDH1* mutational status and gene expression for non-parametric and parametric distributions, respectively. The Mann-Whitney test was also used for functional assays. When the Kruskal-Wallis test was used for gene expression as well as ILS analysis among the different groups and the results were significant, Dunn multiple comparison post hoc test was applied. The Kaplan-Meier survival curves were analyzed using the log rank test (Mantel Cox), excluding 9 cases of tumor relapse in GBM patients. To perform the analysis, the patients were divided into two groups characterized by high (above the median) and low (below the median) gene expression. The statistical significance was set at *p*<0.05. All analyses were performed using SPSS version 15.0 (Chicago, IL), and scatter plots were constructed using the program GraphPad Prism version 5.0 (GraphPad Software, Inc., San Diego, CA).

## Results

### *LOX*, *BMP1* and *HIF1A* Expression Levels in Astrocytomas of Different Malignant Grades

**Fig 1:**
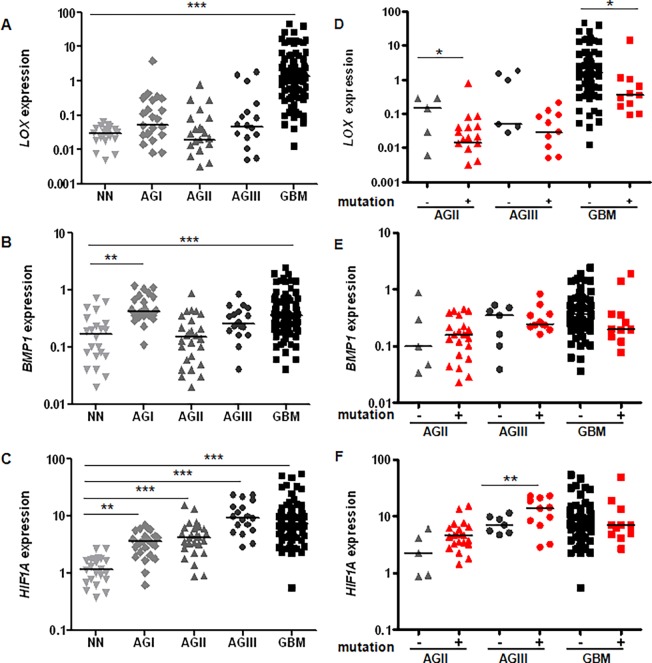
Gene expression levels in astrocytomas of all malignant grades relative to non-neoplastic samples. Transcript levels of *LOX* (A), *BMP1* (B), and *HIF1A* (C) were determined in 19 non-neoplastic brain tissue samples (NN), 23 pilocytic astrocytomas (AGI), 26 low-grade astrocytomas (AGII), 18 anaplastic astrocytomas (AGIII) and 84 glioblastomas (GBM). Total RNA was reverse-transcribed into cDNA and analyzed by quantitative real-time PCR (RT-qPCR) using the SYBR Green method. The differences in expression levels among the groups were statistically significant for *LOX*, *BMP1* and *HIF1A* (Kruskal-Wallis test, *p*<0.0001). A post-hoc Dunn’s test was used to calculate the differences in expression between NN and each astrocytoma group (*p<0.05; **p<0.001; ***p<0.0001). *IDH1* mutational status was determined for diffusely infiltrating cases and the distribution of *LOX* (D), *BMP1* (E) and *HIF1A* (F) expression levels in *IDH1* wild-type and mutated background. Horizontal bars show the median of each group. AGII and GBM cases with *IDH1*-mutated presented lower *LOX* expression levels when compared to *IDH1* wild-type cases. AGIII cases with wild-type *IDH1* presented lower *HIF1A* expression levels when compared to *IDH1*-mutated cases (Mann-Whitney test, * *p* <0.05 and t test **, *p* <0.005).

**Fig 2:**
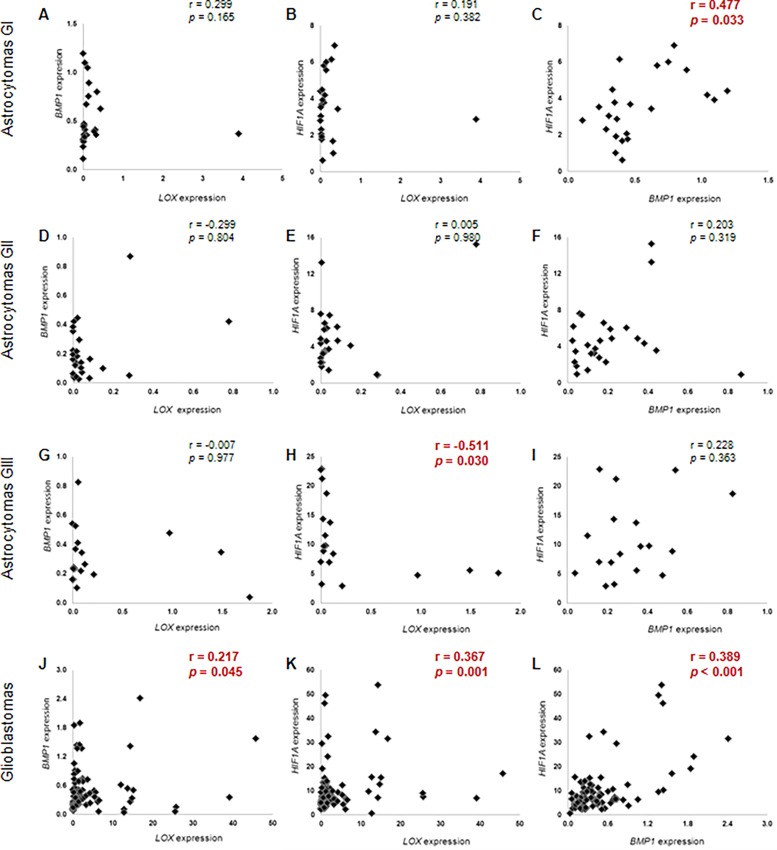
Correlation of *LOX*, *HIF1A* and *BMP1* expression levels in astrocytomas of different malignant grades. Correlation was assessed in pilocytic astrocytomas (AGI: A, B and C), low-grade astrocytomas (AGII: D, E and F), anaplastic astrocytomas (AGIII: G, H and I) and glioblastomas (GBM: J, K and L). Statistically significant values were obtained in AGI cases for *BMP1* and *HIF1A* correlation (C) and in GBM cases for *LOX* and *BMP1* correlation (J), *LOX* and *HIF1A* correlation (K) and *HIF1A* and *BMP1* expression levels (L). In AGIII samples, *HIF1A* and *LOX* expression levels were negatively correlated (H). The statistically significant correlations are shown in red. Correlations between gene expression values were assessed using the non-parametric Spearman-rho correlation test.

*LOX*, *BMP1* and *HIF1A* expression analysis by qRT-PCR showed great variability in astrocytomas of all malignant grades when compared to non-neoplastic samples. The GBM cases had higher *LOX* expression levels relative to non-neoplastic cases, with a statistically significant difference (Fig. 1A). No difference was found in *LOX* expression level between AGI, AGII and AGIII when compared to non-neoplastic samples. On the other hand, *BMP1* expression levels were significantly higher in AGI and GBM groups when compared to non-neoplastic samples (Fig. 1B). *HIF1A* expression levels increased with the malignant grade of astrocytomas, with statistically significant values for all malignant grades of astrocytomas when compared to control samples (Fig. 1C). Coexpression of the three genes was compared for astrocytomas of all malignant grades (Fig. 2). Interestingly, Spearman analysis demonstrated that *LOX* mRNA levels were positively correlated with *BMP1* and *HIF1A* in GBM (*r* = 0.217 and *p* = 0.045, Fig. 2J; *r* = 0.367 and *p* = 0.001, Fig. 2K, respectively). *BMP1* expression also presented a positive correlation with *HIF1A* in GBM (*r* = 0.389; *p*<0.001) (Fig. 2L) and in AGI (*r* = 0.477; *p* = 0.033) (Fig. 2C). *LOX*, *BMP1* and *HIF1A* expression levels were correlated with the clinical outcome of the GBM cases. The analysis of overall survival of GBM cases with high and low expression did not reveal a significant correlation between the gene expression data and the prognosis of GBM patients.

### Association of *LOX*, *BMP1*, and *HIF1A* mRNA Expression Levels and *IDH1* Mutation Status

The frequency of IDH1 mutation was 80.8% in AGII (21 out of 26), 61.1% in AGIII (11 out of 18) and 12.8% in GBM (11 out of 86) (Fig. 1D, 1E and 1F, respectively), which was also described in our previous study of the frequency of *IDH1* mutations in a series of GBM patients [23]. Our GBM cases were composed mainly by primary GBMs, which explains the low frequency of *IDH1* mutation. Of all the mutations, R132H was the most common in glioma. S2 Table summarizes the comparison of the median expression levels of cases with wild-type and mutated *IDH1*. *IDH1*-mutated AGII and GBM cases showed lower *LOX* expression levels when compared to wild-type *IDH1* AGII (*p* = 0.049) and GBM (*p* = 0.008) cases. On the other hand, AGIII cases with wild-type *IDH1* presented lower *HIF1A* expression levels when compared to *IDH1*-mutated cases. (*p* = 0.038). No difference was found for *LOX* expression in AGIII groups with mutated and wild type *IDH1*. Similarly, *BMP1* expression between wild-type and mutated *IDH1* cases was not different regardless of astrocytoma group, and *HIF1A* expression was not different for AGII and GBM.

### Effect of *LOX* Knockdown in GBM Cells

**Fig 3:**
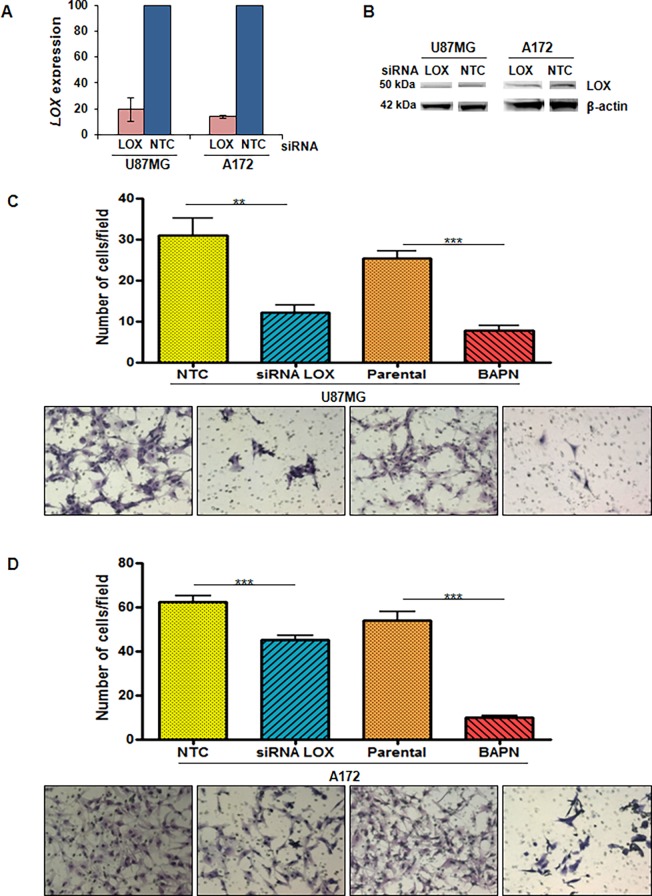
Effect of *LOX* knockdown and inhibition in the migratory phenotype of GBM cell lines. *LOX* expression in U87MG and A172 cell lines transfected with *LOX* siRNA relative to that of cells transfected with the non-targeted control (NTC) siRNA was evaluated 2 days after transfection (A). The data show the average of two independent experiments, and the vertical bar represents the standard deviation. Western blots of LOX protein were analyzed after transfection with siRNA targeted against *LOX* and non-targeted control (NTC) siRNA (B). Evaluation of the migratory behavior of U87MG (C) and A172 (D) cell lines with no treatment (parental), transfected with NTC siRNA and siRNA specific for *LOX* and treated with the LOX inhibitor BAPN. A total of 2.5 x 10^4^ cells were seeded in the upper chamber of Transwell inserts. Cells in the lower chamber were fixed and stained after 18 hours of incubation. Graphs represent the number of migrated cells per field in each condition (means ± standard errors of the means) in two independent experiments. The pictures show representative fields of cells that crossed the insert in all conditions stained with crystal violet at 40x magnification (Mann-Whitney test, **p<0.001; ***p<0.0001).

*LOX* knockdown was evaluated after 2 days of transfection of U87MG and A172 GBM cell lines with siRNA targeted against *LOX* or non-targeted control (NTC) siRNA. The efficiency of transfection was analyzed by RT-qPCR (Fig. 3A) and western blotting (Fig. 3B) for each of the duplicate experiments. Both cell lines presented an approximate 80% decrease in LOX mRNA expression when compared to NTC. Protein expression was also confirmed to be diminished after *LOX* knockdown with siRNA.

**Fig 4:**
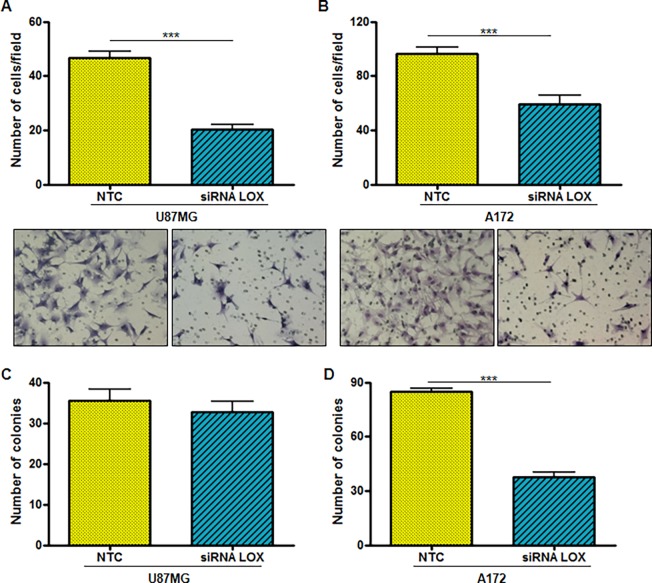
Effect of *LOX* knockdown on the invasive phenotype and anchorage-independent growth behavior of GBM cell lines. Evaluation of the invasion (A and B) and anchorage-independent growth (C and D) of U87MG and A172 cell lines after *LOX* silencing with siRNA were compared to the non-targeted control (NTC). A total of 2.5 x 10^4^ cells were seeded in the upper chamber of Transwell inserts. Cells in the lower chamber were fixed and stained with crystal violet after 18 hours of incubation. Graphs represent the number of migrated cells per field in each condition (means ± standard errors of the means) in the two independent experiments conducted in duplicate. The images show representative fields of U87MG (A) and A172 (B) cells that invaded and crossed the insert (40x magnification). Soft agar colony formation assays were performed for both U87MG (C) and A172 (D) cell lines with 1 x 10^3^ cells. Graphs represent the mean number of colonies ± SD of two independent experiments conducted in duplicate (Mann-Whitney test, **p<0.001; ***p<0.0001).

Transfected cells were maintained under cell culture conditions for 2, 4 and 7 days after transfection to evaluate the involvement of *LOX* in the proliferation of A172 and U87MG cells. There was no difference in the proliferation rate of cells after *LOX* knockdown when compared to NTC in both cell lines analyzed (data not shown). To test the hypothesis that *LOX* expression was correlated with the migratory ability of tumor cells, U87MG and A172 cell lines were evaluated after knocking down *LOX* expression by siRNA and after inhibiting the active form of LOX with a specific drug (BAPN). There was a reduction in the migration ability of U87MG and A172 cells after either transfection with siRNA for LOX or treatment with BAPN, as shown in Fig. 3C and 3D. The differences between the *LOX* siRNA and NTC groups were statistically significant in both U87MG (*p*<0.001) and A172 (*p*<0.0001) cells. Inhibition of LOX by BAPN significantly inhibited the migration of both U87MG and A172 cells when compared to non-treated cells (*p*<0.0001 for both cell lines). LOX involvement in the invasion of GBM cell lines was also observed for U87MG and A172 cells (Fig. 4A and 4B) (*p*<0.0001 for both).

Anchorage-independent growth assays were subsequently used to examine the role of *LOX* in the colony formation ability of U87MG and A172 cells. No reduction in colony numbers was observed for U87MG cells after knockdown of *LOX* when compared to NTC (Fig. 4C). On the other hand, there was a significant decrease in the number of colonies of A172 cells transfected with *LOX* RNAi compared to NTC (*p*<0.0001) (Fig. 4D).

### LOX Immunohistochemistry Analyses

**Fig 5:**
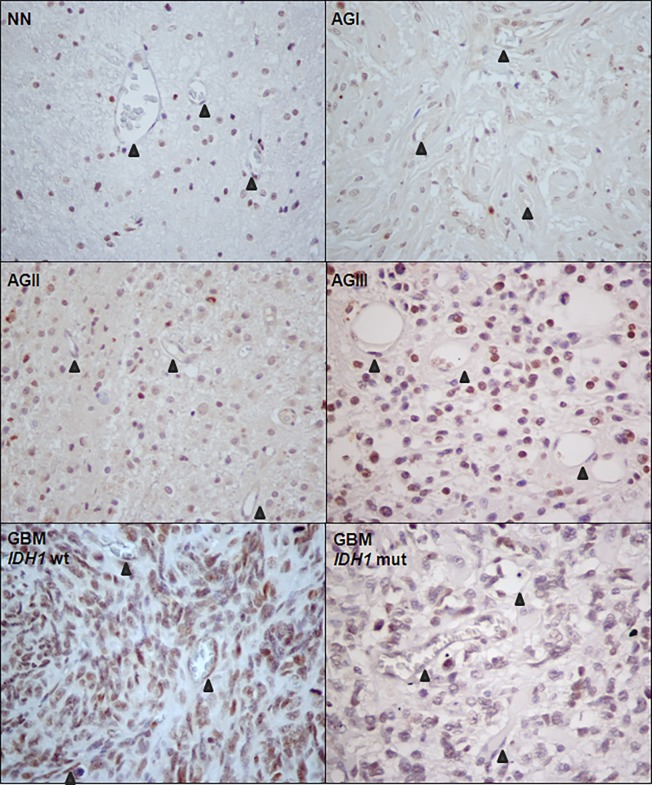
LOX expression and localization in astrocytomas and non-neoplastic brain tissues. Immunohistochemistry was performed in 6 non-neoplastic brain tissues (NN), 6 pilocytic astrocytoma (AGI), 6 low-grade astrocytoma (AGII), 6 anaplastic astrocytoma (AGIII), and 6 glioblastoma (GBM) cases. The images show representative cases of each type of sample (400x magnification for all cases). Arrows indicate the endothelial cells staining.

**Fig 6:**
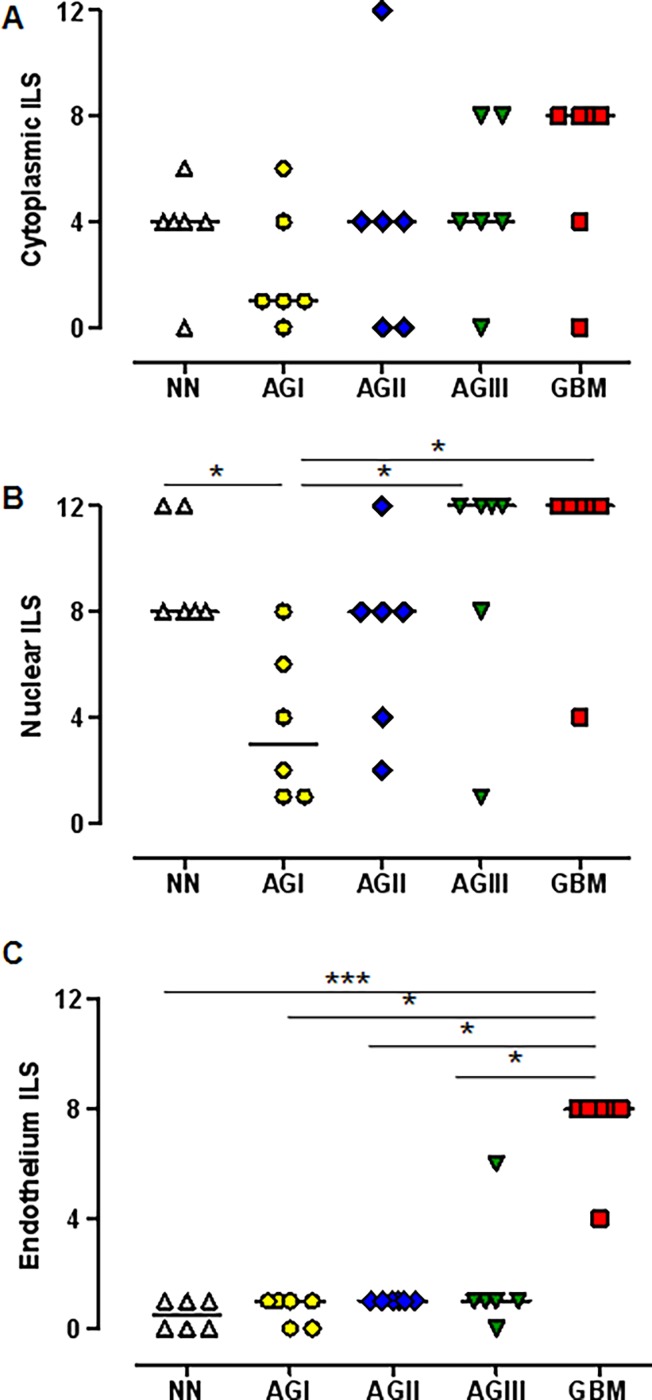
LOX immunostaining analysis in astrocytomas and non-neoplastic brain tissues. The graphs illustrate semi-quantitative immunohistochemistry labeling scores (ILS) that refer to the product of staining intensity and the percentage of positive cells stained with anti-LOX in the cytoplasm (A), nucleus (B) and endothelial cells (C). The horizontal bars show the median ILS of each group. The difference in protein expression among the groups was statistically significant for nuclear staining (*p* = 0.01) and endothelial cell staining (*p* = 0.0008) as determined by a Kruskal-Wallis test. A post-hoc Dunn’s test was used to calculate the differences in expression among the groups (*p<0.05; **p<0.001; ***p<0.0001).

Expression of LOX at the protein level was investigated by immunohistochemistry of non-neoplastic brain tissues and astrocytomas of different malignant grades, as shown in Fig. 5. LOX expression was observed in the cytoplasm and nucleus of all tissues with variable intensity. Endothelial cells were stained specifically in neoformed vessels of GBM cases. The immunohistochemistry results were analyzed semi-quantitatively by the ILS, as described in Methods. The cytoplasmic ILS did not vary significantly among the different astrocytoma groups and control samples (Fig. 6A). Nevertheless, a high nuclear ILS was found in non-neoplastic samples, with staining of glial cells and neurons, while the nuclear ILS in astrocytomas increased with increasing degree of malignancy. AGI group presented statistical differences to non-neoplastic, AGIII and GBM samples (Fig. 6B). Additionally, endothelial cells were predominantly stained for LOX in GBM cases, with significant differences of this tumor group and non-neoplastic and AGI, AGII and AGIII groups (Fig. 6C). Interestingly, GBM cases with mutated *IDH1* presented lower LOX nuclear and endothelial staining, confirming the results of *LOX* transcript expression and *IDH1* mutation status analyses.

## Discussion

In the present study, we investigated the expression of *LOX*, *BMP1* and *HIF1A* in astrocytomas of different malignant grades. Additionally, the effect of *IDH1* mutation on gene expression was also evaluated as well as the role of *LOX* in the behavior of GBM cell lines.

LOX is a secreted amine oxidase that plays a key role in modifying the primary tumor microenvironment by crosslinking collagens and elastin in the ECM [15,25] thereby causing stiffening of the matrix and enhancing the invasive and metastatic properties of the tumor [24, 26, 27, 28]. The stiffness of the ECM is particularly enhanced by the active form of LOX ([15,29], and BMP1 is responsible for processing LOX into the active form [30].

We demonstrated significantly higher *LOX* mRNA expression levels in GBM cases, when compared to non-neoplastic brain tissue samples, as reported by others in different types of tumors [31–35]. Furthermore, higher *BMP1* expression that was positively correlated with *LOX* expression was also detected in GBM cases. Such findings are in agreement with the fact that GBMs are the most malignant and invasive astrocytomas. In fact, we used functional analysis to demonstrate that knocking down *LOX* with siRNA or inhibiting LOX with BAPN led to a reduction in the migration and invasion of U87MG and A172 GBM cell lines. LOX knockdown and BAPN treatment have similar effect on migration in U87MG cell line. On the other hand, the same was not observed for A172 cells even though reduction of LOX expression by siRNA treatment was confirmed. Those results might reflect the GBM heterogeneity. The mutational profile of U87MG and A172 is different according to data of COSMIC cell lines project (http://cancer.sanger.ac.uk/cancergenome/projects/cell_lines/) shows that U87MG has as main consensus genes mutated *PTEN*, *NF1* and *ATRX*, while A172 has *EGFR*, *RB1* and *PTCH1*, among others.

Similar results were obtained by Lackzo et al. [19] using other GBM cells (U251 and U373) treated with BAPN and catalase. These authors associated the role of active LOX in the migration/invasiveness of GBM cell lines with FAK/paxillin activation through hydrogen peroxide generated by LOX catalytic reactions. Intracellular hydrogen peroxide in excess can facilitate the phosphorylation and activation of Src, which then phosphorylates FAK, with consequent induction of various signaling pathways involved in the regulation of cell adhesion and migration [36]. FAK phosphorylation may also explain our finding of a decrease in the number of colonies of A172 cells in which *LOX* expression was knocked down, as suggested by others) [33]. The absence of a similar effect in the colony formation assay with U87MG cells corroborates the heterogeneity observed in this type of tumor.

The irreversible LOX inhibitor BAPN was initially used to treat disorders such as hypertension [37], decreased wound healing [38], and peripheral blood mononuclear cell chemotaxis [39] by preventing the formation of a highly cross-linked form of vascular collagen. Later, BAPN was proposed to also be useful in the treatment of cancers with LOX hyperexpression, such as melanoma [40,41], head and neck carcinoma [42,43] and breast carcinoma [33,44]. Recently, BAPN was used in the treatment of *in vivo* tumor models of pancreatic ductal adenocarcinoma in mice, and it was able to stabilize senescence, delay tumorigenesis, and increase survival [45]. Moreover, another inhibitor of LOX, magnolol, presented a similar effect in a breast cancer model *in vitro* [33]. In addition to BAPN, d-penicillamine, which depletes intracerebral copper, also exhibited antiangiogenic effects on GBM tumor growth in mice [46]. LOX is essential for stimulating endothelial cells and angiogenesis by increasing VEGF expression [20], and the increase in matrix stiffness also upregulates VEGF expression [47]. Interestingly, LOX is expressed in GBM endothelial cells, as demonstrated by immunohistochemistry. This result corroborates a recent report of LOX expression in tumor endothelial cells [41]. The expression of LOX by tumoral and endothelial cells in GBM may be controlled by a positive feedback loop mechanism, although this will require further investigation. Taken together, these results reinforce the notion that LOX may be a target in the treatment of tumors.

The high proliferative capacity of GBM leads to the development of hypoxic areas and ultimately necrosis. The reduction in oxygen availability activates hypoxia-inducible factor-1, which in turn activates the transcription of target genes, including *LOX* [15, 48].

Indeed, our results demonstrated that *HIF1A* is highly expressed in GBM cases, and its expression is associated with *LOX* and *BMP1* expression. Interestingly, the median expression levels of both *LOX* and *BMP1* were higher in AGI samples than in AGII samples. In contrast, the median expression of *HIF1A* in AGI cases was lower than that in AGII. AGI is non-invasive tumor, while AGII is infiltrative. Additionally, AGI presents vascular proliferation and shows greater contrast on neuroimaging compared to AGII. Taking these phenotypic characteristics together, we speculate that *HIF1A* upregulation in AGI activates a hypoxia-driven angiogenic pathway [49]. Additionally, BMP1 can activate various other substrates that are important regulators of extracellular matrix production and quality as well as of antiangiogenic responses by producing a factor from the basal membrane compound [50,51,52,53]. In contrast, stepwise upregulation of *HIF1A* observed in our cases of diffusely infiltrative astrocytomas associated with high expression of *LOX* and *BMP1* suggest progressive activation of both angiogenic and invasive pathways.

Another interesting finding of the present study was the association of *IDH1* mutation with low expression of *LOX*. A list of underexpressed genes responsive to HIF-1, including *LOX*, has been recently reported in *IDH1* mutant gliomas and brain tumor stem cells [21]. In fact, in the present series, *LOX* expression was significantly lower among AGII (p = 0.049) and GBM (p = 0.008) cases presenting *IDH1* mutations when compared to cases with wild-type *IDH1*. For GBM, a decreased level of LOX immunostaining in the nucleus was observed with the presence of *IDH1* mutations. Mutated *IDH1* is a common feature in lower grade gliomas and secondary GBMs [5,7,54,55]. Our series of GBM presented only 12% of IDH1 mutation, consisting mainly of primary cases, as previously reported [56,57]. The presence of mutated *IDH1* is strongly correlated with a CpG island methylator phenotype in gliomas [58]. Therefore, promoter methylation might explain the decreased LOX expression in the presence of such a mutation. Indeed, *LOX* inactivation by methylation has already been demonstrated in gastric cancers as well as colon, lung and ovarian cancer cell lines [59].

Using an antibody against the C-terminus of LOX that detects only the 50 kDa pro-LOX or the processed active 32 kDa LOX, we have unequivocally detected a high level of LOX nuclear staining, particularly in the most malignant astrocytomas (grades III and IV). Previous studies have described only intense perinuclear and cytoplasmic staining of astrocytomas of different malignant grades; nuclear staining has not been mentioned [19]. Intriguingly, nuclear staining was also detected in our normal controls, including glial and neuronal cells, as described by others [60]. These findings were in agreement with previous reports of LOX nuclear localization in smooth muscle [61, 62] and proliferating cells [63]. LOX can oxidize nuclear proteins such as histone H1 [64], leading to epigenetic effects on DNA-histone and histone-histone interactions, with consequent effects on DNA transcription analogous to the effects resulting from histone acetylation [65]. Further studies are needed to clarify differential LOX function in normal and tumor tissues as well as in distinct intracellular compartments.

In summary, our results confirmed that LOX plays an important role in migration and angiogenesis in diffusively infiltrative astrocytomas, especially GBMs. Moreover, *LOX* expression is influenced by *IDH1* mutational status, which provides new insights for the design of targeted therapies to control these tumors.

## Supporting Information

S1 Table Data from samples analyzed in this study. (PDF)

S2 Table Distribution of LOX, BMP1 and HIF1A expression levels in diffusely infiltrative astrocytomas accordingly to IDH1 mutational status. (PDF)
