# Supplementary material for: LOX Expression and Functional Analysis in Astrocytomas and Impact of IDH1 Mutation
Source: PLoS One. 2015 Mar 19;10(3):e0119781. doi: 10.1371/journal.pone.0119781 (PMC4366168; doi:10.1371/journal.pone.0119781)
Supplement: S1 Table — (PDF) [file pone.0119781.s001.pdf]

Supporting Information

Table S1. Data from samples analyzed in this study

Case #	WHO grade[a]	Gene expression[b]			*IDH1* mutation status[c]
			BMP1	***HIF1A***	
1	NN	0.0222	0.2080	0.3711	n.a.
2	NN	0.0071	0.1505	1.2947	n.a.
3	NN	0.0181	0.0225	0.6106	n.a.
4	NN	0.0244	0.0413	0.4481	n.a.
5	NN	0.0186	0.0956	1.1233	n.a.
6	NN	0.0460	0.1973	0.7731	n.a.
7	NN	0.0306	0.0703	1.0139	n.a.
8	NN	0.0542	0.0785	0.9190	n.a.
9	NN	0.0387	0.0747	0.6794	n.a.
10	NN	0.0049	0.0309	0.7729	n.a.
11	NN	0.0077	0.0424	0.4926	n.a.
12	NN	0.0000	0.0800	1.1491	n.a.
13	NN	0.0415	0.3209	2.6671	n.a.
14	NN	0.0211	0.0994	1.7778	n.a.
15	NN	0.0331	0.7267	0.9962	n.a.
16	NN	0.0646	0.6171	1.5513	n.a.
17	NN	0.0332	0.2273	1.7203	n.a.
18	NN	0.0327	0.1998	1.7982	n.a.
19	NN	0.0291	0.1937	1.6491	n.a.
20	NN	0.0501	0.2600	2.7066	n.a.
21	NN	0.0397	0.4927	1.6401	n.a.
22	NN	0.0236	0.4544	1.8687	n.a.
23	AGI	0.0080	0.3060	3.0346	n.a.
24	AGI	0.0914	0.3519	3.7794	n.a.
25	AGI	0.0000	0.1109	2.7833	n.a.
26	AGI	0.0805	0.6689	5.7953	n.a.
27	AGI	0.0296	0.4488	1.7582	n.a.
28	AGI	0.0000	1.1943	4.3959	n.a.
29	AGI	0.0185	0.2854	2.3147	n.a.
30	AGI	0.0471	0.4077	0.6074	n.a.
31	AGI	0.0268	0.4695	3.6660	n.a.
32	AGI	0.1408	0.7536	6.0081	n.a.
33	AGI	0.3251	0.3582	1.0203	n.a.
34	AGI	0.3541	0.7967	6.9023	n.a.
35	AGI	0.4358	0.6249	3.4263	n.a.
36	AGI	0.0140	0.4405	2.0521	n.a.
37	AGI	0.0271	0.3597	1.9181	n.a.
38	AGI	0.0423	0.3353	4.4799	n.a.
39	AGI	0.0083	0.2340	3.5299	n.a.
40	AGI	0.3101	0.4064	1.6597	n.a.
41	AGI	3.9014	0.3683	2.8560	n.a.
42	AGI	0.0507	1.0976	3.9293	n.a.
43	AGI	0.1467	0.8891	5.5615	n.a.
44	AGI	0.1124	1.0435	4.1884	n.a.
45	AGI	0.2799	0.3890	6.1388	n.a.
46	AGII	0.2854	0.8682	0.8682	0
47	AGII	0.0214	0.4442	3.5537	R132H
48	AGII	0.0040	0.4180	13.2361	R132H

49	AGII	0.0000	0.3506	4.8335	R132H
50	AGII	0.0000	0.3841	4.3308	R132H
51	AGII	0.0032	0.0435	1.8001	R132H
52	AGII	0.0000	0.2207	4.8403	R132H
53	AGII	0.0290	0.0227	4.6023	R132H
54	AGII	0.2812	0.0467	0.9072	0
55	AGII	0.0000	0.1583	2.7527	R132H
56	AGII	0.1477	0.0985	4.1014	0
57	AGII	0.0062	0.0336	2.2322	0
58	AGII	0.0000	0.0599	7.6080	R132H
59	AGII	0.0442	0.0699	7.4452	R132H
60	AGII	0.0811	0.0286	6.1717	R132H
61	AGII	0.0137	0.0396	3.4246	R132H
62	AGII	0.0141	0.1326	3.2505	R132H
63	AGII	0.0200	0.1805	6.5663	R132H
64	AGII	0.0300	0.2932	6.0209	0
65	AGII	0.0096	0.1182	3.2020	R132H
66	AGII	0.0396	0.1383	3.7342	R132H
67	AGII	0.0000	0.1913	2.2566	R132H
68	AGII	0.7789	0.4189	15.2379	R132H
69	AGII	0.0845	0.1616	4.6278	R132H
70	AGII	0.0177	0.2131	5.8945	R132H
71	AGII	0.0400	0.0989	1.3867	R132H
72	AGIII	0.0421	0.1011	11.4578	0
73	AGIII	0.0054	0.1610	22.8621	R132H
74	AGIII	0.0000	0.5416	22.7126	R132H
75	AGIII	0.0218	0.2334	14.3286	R132H
76	AGIII	0.2142	0.1930	2.8422	R132H
77	AGIII	0.0105	0.2440	21.1821	R132H
78	AGIII	1.7762	0.0388	5.0941	0
79	AGIII	0.0270	0.5267	8.8459	0
80	AGIII	0.0496	0.4107	9.7210	0
81	AGIII	0.0288	0.3672	9.6438	R132H
82	AGIII	0.0813	0.2199	6.9163	R132H
83	AGIII	0.1239	0.2656	8.3837	R132H
84	AGIII	0.0050	0.2349	3.2048	R132H
85	AGIII	0.9697	0.4765	4.7098	0
86	AGIII	0.0543	0.8276	18.6621	R132H
87	AGIII	1.4920	0.3480	5.5492	0
88	AGIII	0.0905	0.3449	13.7293	R132H
89	AGIII	0.0000	0.1605	6.9694	0
90	GBM	1.4273	0.5152	4.9702	0
91	GBM	0.0881	0.0998	4.4098	0
92	GBM	12.8551	0.1080	15.6628	0
93	GBM	1.6620	0.6820	6.1814	0
94	GBM	3.3268	0.1940	4.2993	0
95	GBM	2.4642	0.7428	6.5710	0
96	GBM	5.3860	0.4928	8.1354	0
97	GBM	0.4699	0.3686	9.1279	0
98	GBM	0.5712	0.2926	9.5608	0
99	GBM	0.0523	0.2152	2.2021	0
100	GBM	0.0572	0.1020	4.1888	0
101	GBM	1.8676	0.4834	8.2033	0
102	GBM	0.5977	0.9123	3.6873	0

103	GBM	1.4190	0.3763	3.6549	0
104	GBM	2.2822	1.3665	9.4837	0
105	GBM	0.0971	0.0759	2.6490	R132H
106	GBM	5.7095	0.4517	4.3874	0
107	GBM	1.2000	0.9032	12.4069	0
108	GBM	0.7708	0.4946	8.7808	0
109	GBM	25.7978	0.1465	7.3829	0
110	GBM	0.3198	0.1359	7.5696	0
111	GBM	0.5138	0.5584	2.6379	0
112	GBM	1.0050	1.4361	46.1152	0
113	GBM	0.0394	0.1119	2.7524	0
114	GBM	2.5182	0.5043	8.3246	0
115	GBM	4.3321	0.2293	9.2218	0
116	GBM	0.2860	0.1900	4.7872	R132H
117	GBM	1.0041	0.3312	15.1461	0
118	GBM	14.3708	0.2624	6.9889	R132H
119	GBM	0.3608	0.2123	3.7557	0
120	GBM	13.7585	0.5405	34.2314	0
121	GBM	0.5451	0.6482	4.8554	0
122	GBM	2.3607	0.7043	7.2562	0
123	GBM	0.1604	0.1195	4.1272	R132H
124	GBM	1.9147	0.3821	9.3641	0
125	GBM	0.3657	0.8343	6.2060	0
126	GBM	1.8440	0.3386	10.9496	0
127	GBM	25.6746	0.0626	8.9214	0
128	GBM	0.1042	0.2731	2.5894	0
129	GBM	0.7501	0.2483	5.2062	0
130	GBM	0.8691	0.2055	2.8829	0
131	GBM	1.8432	0.3213	32.3833	0
132	GBM	15.1733	0.4995	15.4386	0
133	GBM	1.4469	0.2085	13.1129	0
134	GBM	0.0123	0.5097	2.2779	0
135	GBM	12.0624	0.6081	9.7300	0
136	GBM	1.6341	1.4424	10.2213	0
137	GBM	0.3399	0.7311	29.4074	0
138	GBM	2.5945	0.6880	6.3444	0
139	GBM	0.9223	0.1886	2.7101	0
140	GBM	1.1881	0.2421	11.0703	0
141	GBM	2.4196	0.3153	10.7759	0
142	GBM	3.6669	0.7192	6.5186	0
143	GBM	0.3155	1.0576	6.3899	0
144	GBM	1.1390	1.3687	49.4465	R132H
145	GBM	14.4176	1.4140	53.6221	0
146	GBM	16.8332	2.4170	31.5210	0
147	GBM	0.3545	1.8515	19.0098	R132H
148	GBM	1.7909	1.8996	24.0120	0
149	GBM	6.2422	0.2655	2.2611	0
150	GBM	0.4027	0.1952	7.0754	R132H
151	GBM	12.7773	0.0353	0.5436	0
152	GBM	0.1198	0.2560	9.3438	0
153	GBM	2.9550	0.3543	9.9394	0
154	GBM	0.1968	0.3684	6.1667	R132H
155	GBM	45.7766	1.5708	16.9891	0
156	GBM	1.5908	0.4553	4.9410	0

157	GBM	0.5750	0.2327	13.2837	0
158	GBM	1.4096	0.6832	10.5951	0
159	GBM	3.3510	0.3949	6.5869	0
160	GBM	0.2648	0.4547	11.2206	0
161	GBM	0.9051	0.2663	3.0128	0
162	GBM	1.2767	0.2917	9.6293	0
163	GBM	0.2989	0.1687	5.8263	0
164	GBM	5.4582	0.3669	12.2816	0
165	GBM	0.1177	0.1821	5.4194	0
166	GBM	39.1774	0.3553	6.9497	0
167	GBM	14.8511	0.3397	12.6188	0
168	GBM	0.0913	0.1452	5.0503	R132H
169	GBM	1.0054	0.1965	13.5267	R132H
170	GBM	1.4867	0.2574	13.8525	0
171	GBM	0.6439	0.3475	7.0610	R132H
172	GBM	6.4639	0.0582	6.4415	0
173	GBM	1.9512	0.4778	7.6442	0
174	GBM	4.2372	0.4393	5.6105	0
175	GBM	6.6109	0.2852	6.2111	0

[a]NN, non-neoplastic; AGI, pilocytic astrocytoma; AGII, low-grade astrocytoma; AGIII, anaplastic astrocytoma; GBM, glioblastoma

[b]*LOX*, lysyl oxidase gene; *BMP1*: bone morphogenetic protein-1; *HIF1A*: factor induced hypoxia-induced factor 1-alpha gene

[c]0, no mutation; n.a., not analyzed

Table S2. Distribution of LOX, BMP1 and HIF1A expression levels in diffusely infiltrative astrocytomas accordingly to IDH1 mutational status

		IDH1		
	Genes	wild-type	mutated	p
AGII	*LOX*	0.148 (0.006 - 0.2854)	0.014 (0.000 - 0.779)	**0.049***
	BMP1	2.232 (0.868 - 6.021)	4.602 (1.436- 15.238)	0.850*
	HIF1A	0.099 (0.034 - 0.868)	0.158 (0.023 - 0.444)	0.091*
	n (%)	5 (19.2)	21 (80.8)	
AGIII	*LOX*	0.050 (0.027 - 1.776)	0.029 (0.005 - 0.214)	0.285*
	BMP1	6.969 (4.710 - 11.458)	13.729 (2.842 - 22.862)	0.791*
	HIF1A	0.348 (0.039 - 0.527)	0.244 (0.161 - 0.828)	**0.038****
	n (%)	7 (38.9)	11 (61.1)	
GBM	*LOX*	1.634 (0.012 - 45.777)	0.355 (0.091 - 14.371)	**0.008***
	BMP1	7.644 (0.544 - 53.622)	6.989 (2.65 - 49.447)	0.119*
	HIF1A	0.369 (0.035 - 2.417)	0.197 (0.076 - 1.852)	0.742*
	n (%)	75 (87.2)	11 (12.8)	

*Mann-Whitney test, **t test. In bold, the statistically signficant values

Gene expression levels: median (minimun - maximum)
