# Supplementary material for: LOX Expression and Functional Analysis in Astrocytomas and Impact of IDH1 Mutation
Source: PLoS One. 2015 Mar 19;10(3):e0119781. doi: 10.1371/journal.pone.0119781 (PMC4366168; doi:10.1371/journal.pone.0119781)
Supplement: S2 Table — (PDF) [file pone.0119781.s002.pdf]

Table S2. Distribution of LOX, BMP1 and HIF1A expression levels in diffusely infiltrative astrocytomas accordingly to IDH1 mutational status

		IDH1		
	Genes	wild-type	mutated	p
AGII	*LOX*	0.148 (0.006 - 0.2854)	0.014 (0.000 - 0.779)	**0.049***
	BMP1	2.232 (0.868 - 6.021)	4.602 (1.436- 15.238)	0.850*
	HIF1A	0.099 (0.034 - 0.868)	0.158 (0.023 - 0.444)	0.091*
	n (%)	5 (19.2)	21 (80.8)	
AGIII	*LOX*	0.050 (0.027 - 1.776)	0.029 (0.005 - 0.214)	0.285*
	BMP1	6.969 (4.710 - 11.458)	13.729 (2.842 - 22.862)	0.791*
	HIF1A	0.348 (0.039 - 0.527)	0.244 (0.161 - 0.828)	**0.038****
	n (%)	7 (38.9)	11 (61.1)	
GBM	*LOX*	1.634 (0.012 - 45.777)	0.355 (0.091 - 14.371)	**0.008***
	BMP1	7.644 (0.544 - 53.622)	6.989 (2.65 - 49.447)	0.119*
	HIF1A	0.369 (0.035 - 2.417)	0.197 (0.076 - 1.852)	0.742*
	n (%)	75 (87.2)	11 (12.8)	

*Mann-Whitney test, **t test. In bold, the statistically signficant values

Gene expression levels: median (minimun - maximum)
